# Supplementary material for: Contribution of tree community structure to forest productivity across a thermal gradient in eastern Asia
Source: Nat Commun. 2023 Mar 13;14:1113. doi: 10.1038/s41467-023-36671-1 (PMC10011560; doi:10.1038/s41467-023-36671-1)
Supplement: Supplementary file 1 — Supplementary information [file 41467_2023_36671_MOESM1_ESM.pdf]

Supplementary information

Forest productivity across a thermal gradient in eastern Asia reflects tree community structure

Tetsuo I. Kohyama, Douglas Shei, I-Fang Sun, Kaoru Niiyama, Eizi Suzuki, Tsutom Hiura, Naoyuki Nishimura, Kazuhiko Hoshizaki, Shu-Hui Wu, Wei-Chun Chao, Zamah S. Nur Hajar, Joeni S. Rahajoe & Takashi S. Kohyama

Corresponding author: tetsuo.kohyama@gmail.com

Supplementary figures 1–8

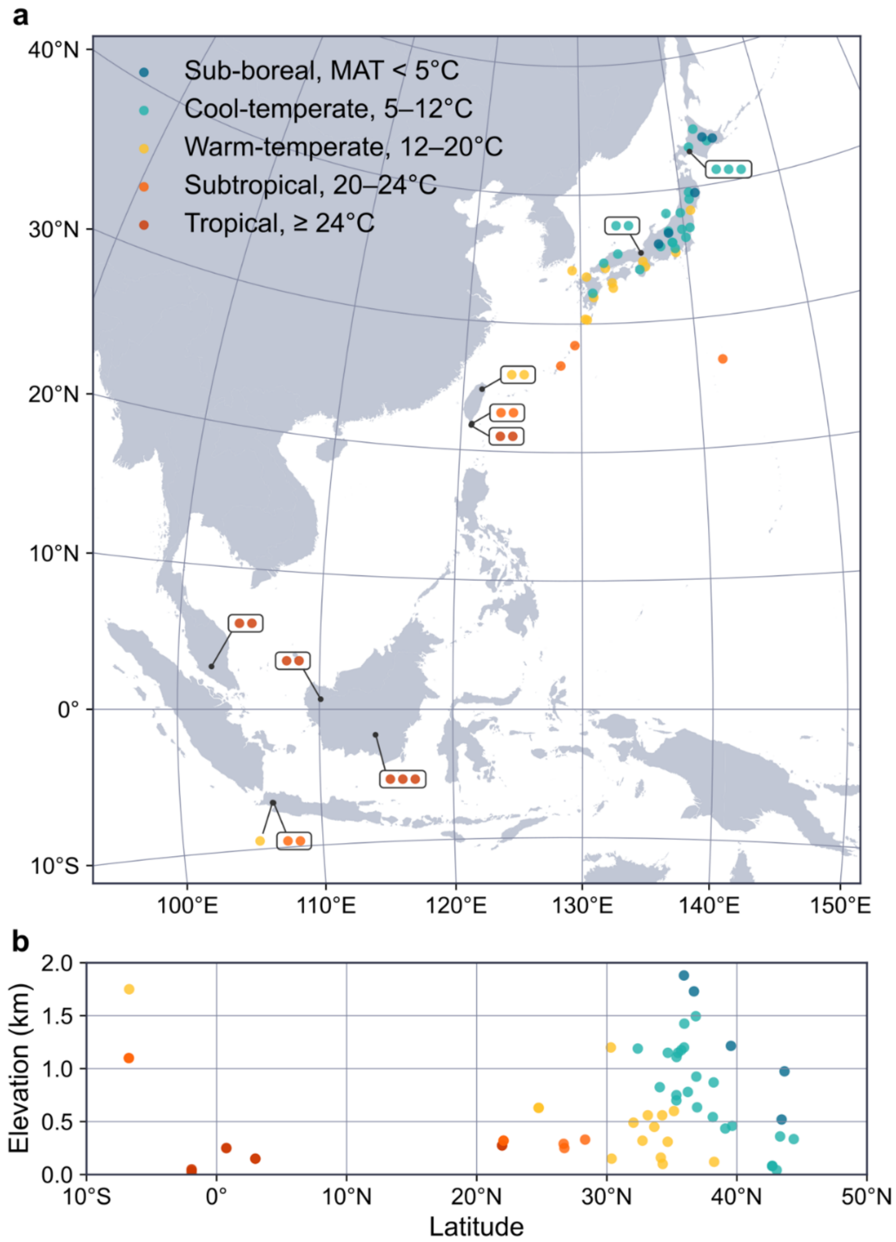

Supplementary Fig. 1. Geographic distribution of 60 forest plots in eastern Asia. Circles show forest plots studied, of which colours indicate mean annual temperature (MAT). **a** Geographic map. The map was created using free vector and raster map data from Natural Earth (https://www.naturalearthdata.com). **b** Plot distribution on latitude-elevation coordinates.

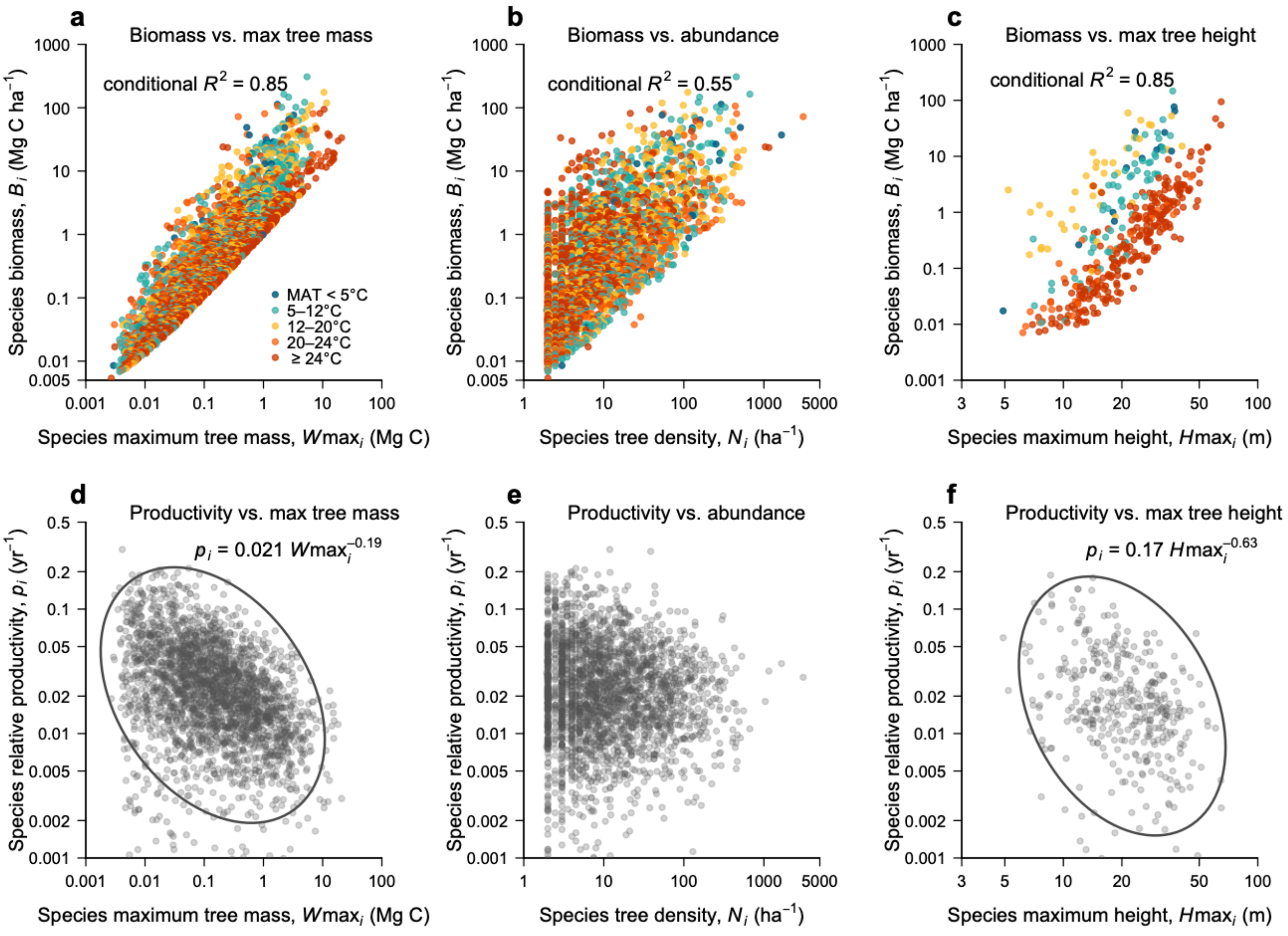

Supplementary Fig. 2. Determinants of species aboveground biomass and their contribution to species relative woody productivity. a Species aboveground biomass against maximum aboveground individual tree mass of species; **b** species biomass against tree abundance (per-ha tree density); **c** species biomass against tallest tree height of species; **d** species relative aboveground woody productivity against largest tree aboveground mass; **e** species relative woody productivity against tree abundance; **f** species relative woody productivity against species' tallest tree height. The conditional coefficient of determination R^2 for each pairwise log-log linear model is shown in **a**–**c**; the fit models where the model constant term is dependent on plot identity (as a mixed effect). The multiple log-linear model of species biomass by the largest tree mass and abundance combined shows $R^2 = 0.96$. The 95% log-log normal distribution range is shown in **d** and **f**, but not **e** where the sign of regression coefficient (i.e. power-law exponent) is negative at –0.05 by simple log-linear model fit and positive at 0.10 by multiple log-linear model fit with maximum tree mass.

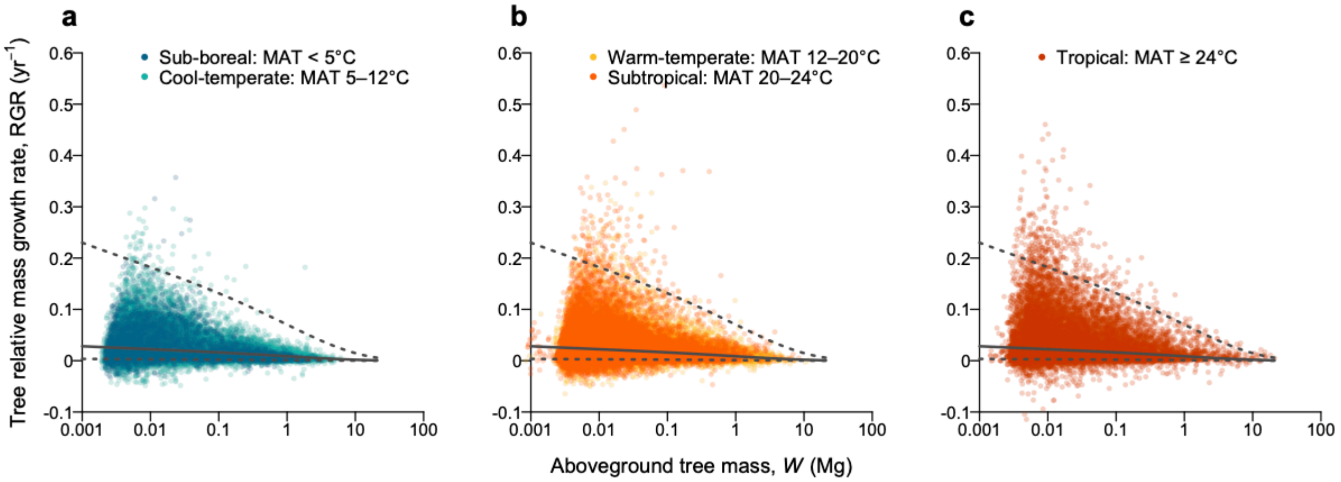

Supplementary Fig. 3. Relationships between tree relative mass growth rate and tree aboveground mass across all species populations of 60 plots in eastern Asia. Tree relative mass growth rate, RGR, is $\ln(W_T/W_0)$ / T (yr^{-1}), and mean tree mass, W, is $(W_T - W_0)$ / $\ln(W_T/W_0)$, where W_0 and W_T is tree mass (Mg C) at first and second census, respectively, and T (yr) is inter-census interval. Among all surviving trees in all plots, we excluded those with $\ln(W_{T/.}/W_0) < -0.1$, supposing mismeasurements[42] (resulting in 72,856 trees in total). Regression curve (expectation in full line, 95% prediction interval by dashed lines) is shown for all trees pooled, excluding trees with non-positive RGR[42] as:

$$\ln \mathrm{RGR} = -4.1 - 0.082 \ln W - 0.64\, W^{0.5}.$$

When we include mean annual temperature (MAT) as an explanatory variable,

$$\ln \mathrm{RGR} = -4.3 - 0.081 \ln W - 0.61\, W^{0.5} + 0.014\, \mathrm{MAT},$$

where the MAT-dependence coefficient of 0.014±0.001°C^{-1} is not significantly different from that of 0.018±0.006°C^{-1} in our regression model of forest level productivity for the simulated data productivity P_{specRes} for the species response hypothesis (Fig. 4b).

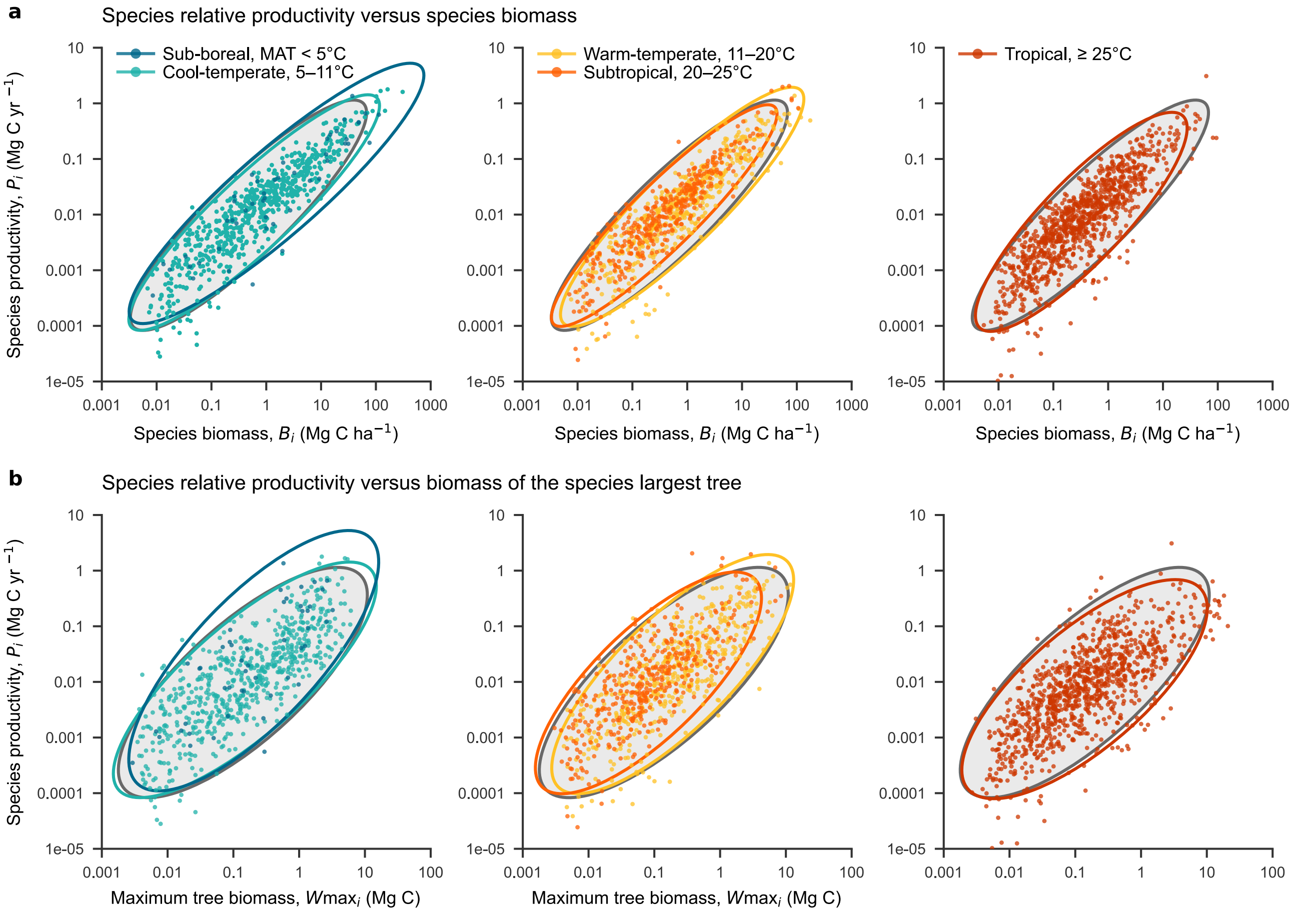

Supplementary Fig. 4. Species-level standing biomass and absolute woody productivity across 2,665 species populations in 60 forest plots in eastern Asia. Plots are grouped by mean annual temperature (MAT). **a** Relationships between per-plot species-*i* absolute aboveground woody productivity P_i, against species aboveground biomass B_i, and **b** those between species absolute productivity P_i against species' maximum tree mass, $W\max_i$, on log-log scale. The 95% log-normal prediction ellipses are shown for each of five biomes grouped by MAT by corresponding colours, and the common ellipse for all species populations in all plots by filled grey.

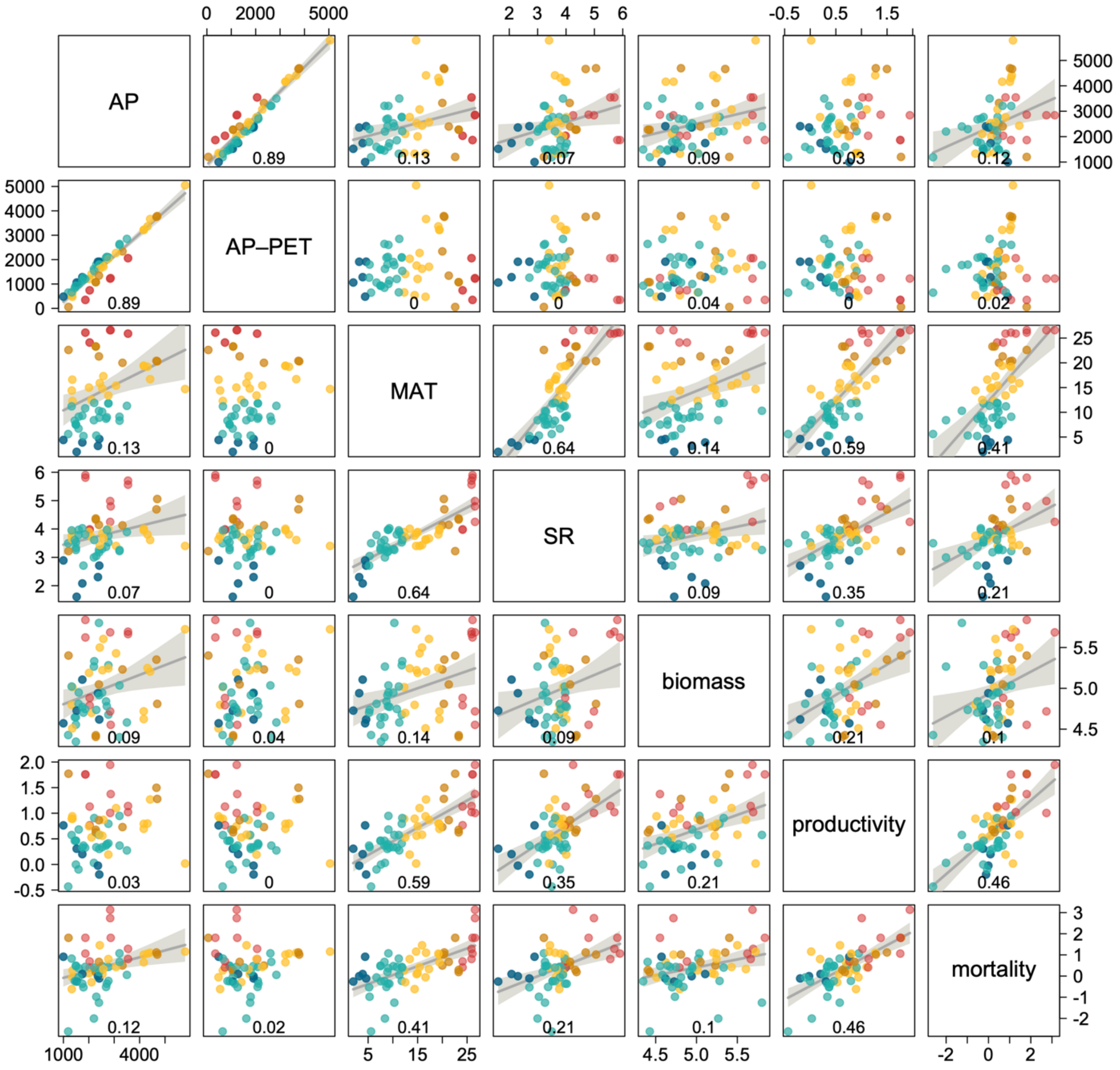

Supplementary Fig. 5. Pairwise regression diagrams between variables among 60 forest plots in eastern Asia. ‘AP’, annual precipitation (mm yr^{-1}); ‘AP–PET’, annual precipitation minus annual potential evapotranspiration (mm yr^{-1}); ‘MAT’, mean annual temperature (°C); ‘SR’, the number of species per plot; ‘biomass’, forest level per-area aboveground biomass (Mg C ha^{-1}); ‘productivity’, forest aboveground woody primary productivity (Mg C ha^{-1} yr^{-1}); ‘loss rate’, forest aboveground woody loss rate (Mg C ha^{-1} yr^{-1}). SR, biomass, productivity and loss rate are natural-log transformed. Symbol colours indicate MAT of forest plots as in Figs. 2, 4 and Supplementary Fig. 1. The regression line indicates mean and 95% confidence intervals predicted by the linear model and is displayed if the probability (i.e. the two-tailed statistical significance of Pearson's correlation coefficient) is less than 0.05. Number of plots are 60 and the degrees of freedom are 58. Inset values show the coefficient of determination (R^2).

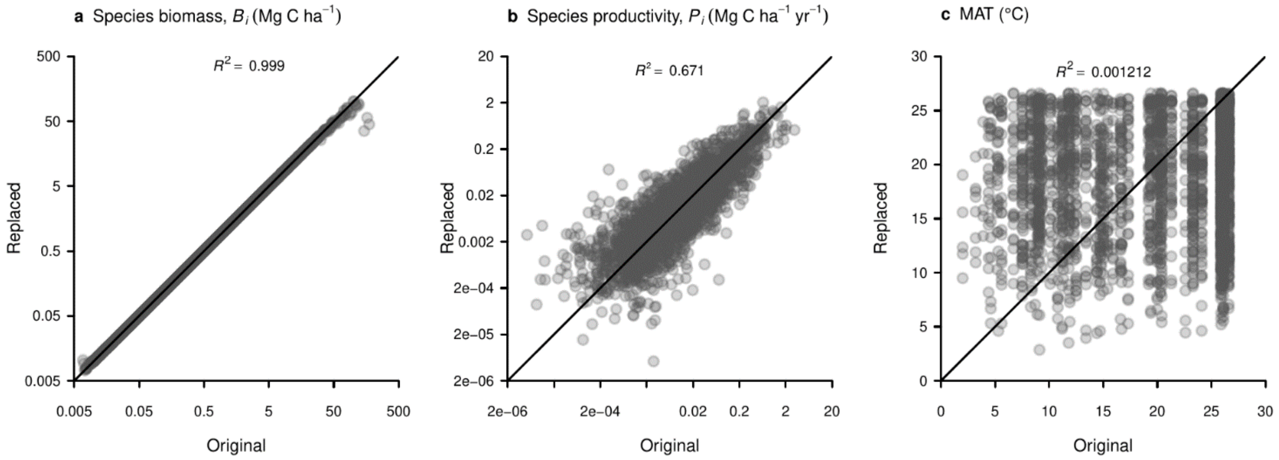

Supplementary Fig. 6. Comparison of original and replaced data. **a** species aboveground biomass B_i, **b** species aboveground woody productivity P_i, and **c** mean annual temperature MAT of the forest plot from which the tree was sampled. Each point represents a species population. A straight line with an intercept of 0 and a slope of 1 is shown. **a** and **b** are on log-log scale; **c** on a normal scale.

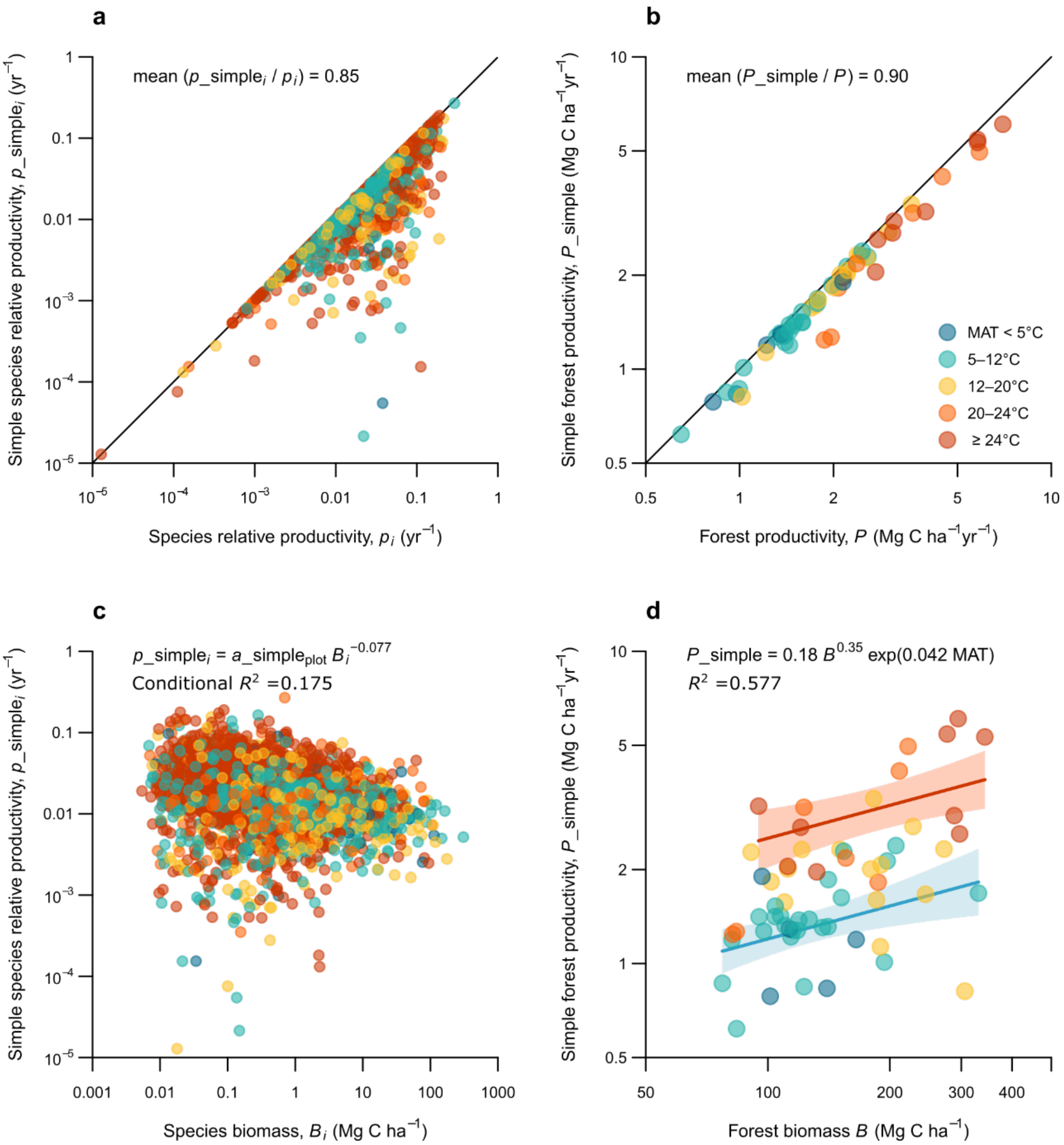

Supplementary Fig. 7. Biases of standard woody productivity estimates. a Comparison of species level simple relative aboveground woody productivity, p_simple_i, by standard estimation[17] against instantaneous aboveground woody productivity, p_i. **b** Forest plot level simple aboveground woody productivity, *P*_simple, against instantaneous aboveground woody productivity *P*. **c** Species level simple woody productivity p_simple_i against species aboveground biomass B_i. Plot-identity-dependent constant coefficient estimated by the power-law model (inset), a_simple_{plot} ranges [0.0086, 0.045]. **d** Forest plot level simple woody productivity *P*_simple against plot aboveground biomass *B*. Lines and bands indicate mean and 95% confidence intervals predicted by the power-law model at tropical (> 24°C, red line) versus cool-temperate/sub-boreal forests (< 12°C, blue lines). In **a** and **c**, only species populations with positive p_simple_i are shown (2,465 out of 2,604 populations). Unlike our procedure of obtaining *P* (= $\Sigma_i p_i B_i$), p_simple_i is obtained by partitioning forest level *P*_simple into each population, then divided by population biomass B_i.The number of observations for **b** and **d** are 60.

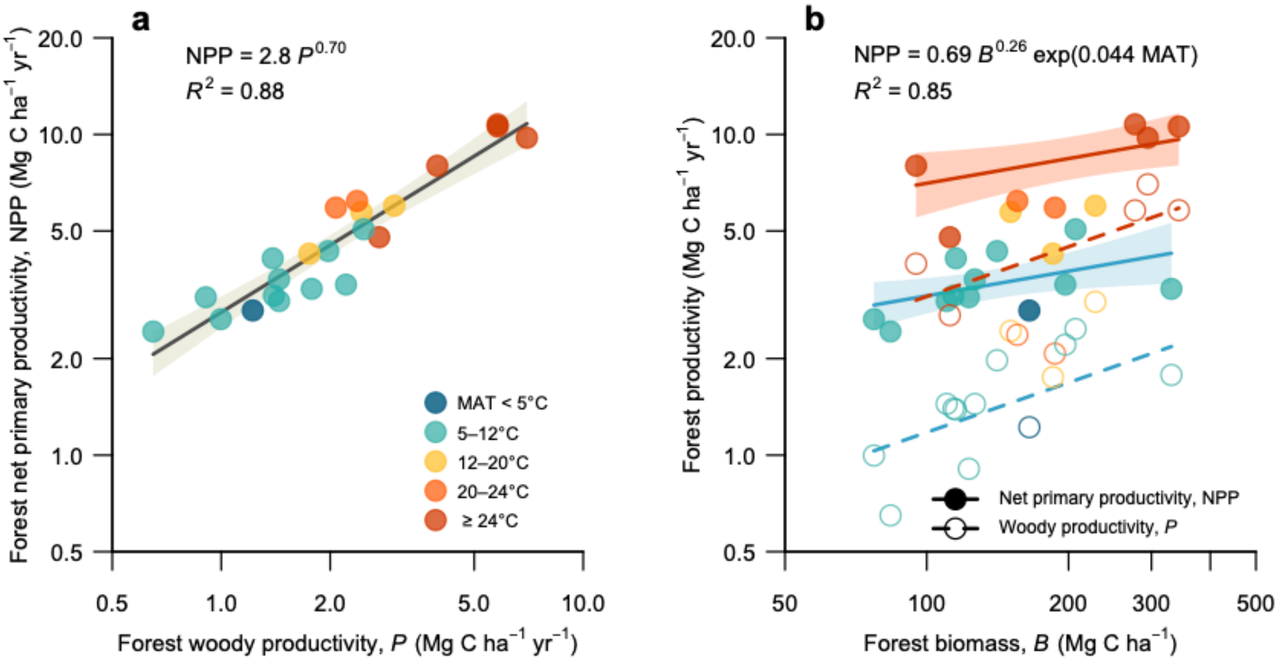

Supplementary Fig. 8. Relationship of forest-level woody productivity, net primary productivity, and standing biomass among 22 forest plots in eastern Asia. a Aboveground net primary productivity (NPP) against aboveground woody productivity *P*. **b** Aboveground net primary productivity and woody productivity against aboveground biomass. Colours indicate forest biome classified by mean annual temperature (MAT). Regression lines indicates mean and 95% confidence intervals predicted by power law model. In **b**, MAT-dependence is shown for tropical (> 24°C, red line) versus cool-temperate/sub-boreal forests (< 12°C, blue lines); open circles and broken lines indicate woody productivity, *P*. Aboveground net primary productivity is obtained by *P* plus canopy productivity from monthly fine litter fall records.
