# Supplementary material for: Contribution of tree community structure to forest productivity across a thermal gradient in eastern Asia
Source: Nat Commun. 2023 Mar 13;14:1113. doi: 10.1038/s41467-023-36671-1 (PMC10011560; doi:10.1038/s41467-023-36671-1)
Supplement: Supplementary file 2 — Reporting Summary [file 41467_2023_36671_MOESM2_ESM.pdf]

nature portfolio

Corresponding author(s): Tetsuo Kohyama

Last updated by author(s): Feb 10, 2023

Reporting Summary

Nature Portfolio wishes to improve the reproducibility of the work that we publish. This form provides structure for consistency and transparency in reporting. For further information on Nature Portfolio policies, see our Editorial Policies and the Editorial Policy Checklist.

Statistics

For all statistical analyses, confirm that the following items are present in the figure legend, table legend, main text, or Methods section.

n/a	Confirmed	
☐	☒	The exact sample size (*n*) for each experimental group/condition, given as a discrete number and unit of measurement
☐	☒	A statement on whether measurements were taken from distinct samples or whether the same sample was measured repeatedly
☐	☒	The statistical test(s) used AND whether they are one- or two-sided<br>*Only common tests should be described solely by name; describe more complex techniques in the Methods section.*
☒	☐	A description of all covariates tested
☒	☐	A description of any assumptions or corrections, such as tests of normality and adjustment for multiple comparisons
☐	☒	A full description of the statistical parameters including central tendency (e.g. means) or other basic estimates (e.g. regression coefficient) AND variation (e.g. standard deviation) or associated estimates of uncertainty (e.g. confidence intervals)
☐	☒	For null hypothesis testing, the test statistic (e.g. *F*, *t*, *r*) with confidence intervals, effect sizes, degrees of freedom and *P* value noted<br>*Give P values as exact values whenever suitable.*
☒	☐	For Bayesian analysis, information on the choice of priors and Markov chain Monte Carlo settings
☐	☒	For hierarchical and complex designs, identification of the appropriate level for tests and full reporting of outcomes
☒	☐	Estimates of effect sizes (e.g. Cohen's *d*, Pearson's *r*), indicating how they were calculated

Our web collection on statistics for biologists contains articles on many of the points above.

Software and code

Policy information about availability of computer code

Data collection	No software was used.
Data analysis	R version 4.0.5 and Python version 3.9 were used. The code used in this study is available in the Zenodo repository (https://doi.org/10.5281/zenodo.7370509).

For manuscripts utilizing custom algorithms or software that are central to the research but not yet described in published literature, software must be made available to editors and reviewers. We strongly encourage code deposition in a community repository (e.g. GitHub). See the Nature Portfolio guidelines for submitting code & software for further information.

Data

Policy information about availability of data

All manuscripts must include a data availability statement. This statement should provide the following information, where applicable:

- Accession codes, unique identifiers, or web links for publicly available datasets
- A description of any restrictions on data availability
- For clinical datasets or third party data, please ensure that the statement adheres to our policy

Full datasets at the level of all trees and those compiled for species populations have been deposited in the Zenodo repository (https://doi.org/10.5281/zenodo.7370509). The original data of forest plots in Japan is available at is available at https://www.biodic.go.jp/moni1000/findings/data/ (version June 2019). Climate data used in this study, CHELSA version 2.1, is available at https://chelsa-climate.org/.

Human research participants

Policy information about studies involving human research participants and Sex and Gender in Research.

Reporting on sex and gender	Not applicable
Population characteristics	Not applicable
Recruitment	Not applicable
Ethics oversight	Not applicable

Note that full information on the approval of the study protocol must also be provided in the manuscript.

Field-specific reporting

Please select the one below that is the best fit for your research. If you are not sure, read the appropriate sections before making your selection.

☐ Life sciences ☐ Behavioural & social sciences ☒ Ecological, evolutionary & environmental sciences

For a reference copy of the document with all sections, see nature.com/documents/nr-reporting-summary-flat.pdf

Ecological, evolutionary & environmental sciences study design

All studies must disclose on these points even when the disclosure is negative.

Study description	Cross-regional comparison of forest productivity based on repeated census data from a total of 88,587 trees in 60 old-growth forest plots in Japan, Taiwan, Malaysia, and Indonesia.
Research sample	All trees in 60 forest plots were used to estimate forest productivity. Forest plot data were collected from published literature and personal archives of the co-authors, as well as from existing databases (https://www.biodic.go.jp/moni1000/findings/data/).
Sampling strategy	All trees with a diameter width of 5 cm or greater in 1 ha forest plots were used in the analysis. No sample size calculations are performed prior to analysis. The sample size was considered to have sufficient spatial coverage compare to that used in the previous studies in forest productivity.
Data collection	Stem size records for all trees in each of 60 forest plots were collected from published literature and personal archives, as well as from existing databases. The initial collection of data was done by all co-authors, the data compilation was done by the corresponding author.
Timing and spatial scale	Data were collected in 60 old-growth forest plots (1 ha each) set from 6.8°S–44.4°N latitude in eastern Asia, with two censuses conducted between 1992 and 2016. To minimize bias in estimating forest productivity, the dataset with an interval between two censuses of about five years was chosen for each plot.
Data exclusions	No data were excluded.
Reproducibility	This is not an experimental study, thus experimental replication was not performed. The analysis is reproducible given the code provided, which includes all steps from raw data to end results.
Randomization	All trees in each plots are sampled; multiple plots in each forest type; statistical randomization was carried out to test two hypotheses.
Blinding	Not relevant, since this study is not experimental.

Did the study involve field work? ☐ Yes ☒ No

Reporting for specific materials, systems and methods

We require information from authors about some types of materials, experimental systems and methods used in many studies. Here, indicate whether each material, system or method listed is relevant to your study. If you are not sure if a list item applies to your research, read the appropriate section before selecting a response.

Materials & experimental systems

n/a	Involved in the study	
☒	☐	Antibodies
☒	☐	Eukaryotic cell lines
☒	☐	Palaeontology and archaeology
☒	☐	Animals and other organisms
☒	☐	Clinical data
☒	☐	Dual use research of concern

Methods

n/a	Involved in the study	
☒	☐	ChIP-seq
☒	☐	Flow cytometry
☒	☐	MRI-based neuroimaging
