# Supplementary material for: Contribution of tree community structure to forest productivity across a thermal gradient in eastern Asia
Source: Nat Commun. 2023 Mar 13;14:1113. doi: 10.1038/s41467-023-36671-1 (PMC10011560; doi:10.1038/s41467-023-36671-1)
Supplement: Supplementary file 3 — Description of Additional Supplementary Files [file 41467_2023_36671_MOESM3_ESM.pdf]

File Name: Supplementary Data 1
Description: Location and census years of 60 forest plots examined.

File Name: Supplementary Data 2
Description: Climate data for 60 forest plots.

File Name: Supplementary Data 3
Description: Community and ecosystem variables of 60 forest plots examined.
